# Prevalence and Clinical Impact of Electrocardiographic Abnormalities in Patients with Chronic Kidney Disease

**DOI:** 10.3390/jcm11185414

**Published:** 2022-09-15

**Authors:** Sejun Park, Yunjin Yum, Jung-Joon Cha, Hyung Joon Joo, Jae Hyoung Park, Soon Jun Hong, Cheol Woong Yu, Do-Sun Lim

**Affiliations:** 1 Department of Internal Medicine, Korea University Anam Hospital, Seoul 02841, Korea; 2 Department of Biostatistics, Korea University College of Medicine, Seoul 02841, Korea; 3 Department of Internal Medicine, Division of Cardiology, Korea University Anam Hospital, Seoul 02841, Korea; 4 Department of Medical Informatics, Korea University College of Medicine, Seoul 02841, Korea; 5 Research Institute for Medical Bigdata Science, College of Medicine, Korea University, Seoul 02708, Korea

**Keywords:** electrocardiogram, chronic kidney disease, major adverse cerebrocardiovascular events

## Abstract

Chronic kidney disease (CKD) is a strong risk factor for cardiovascular disease. An electrocardiogram (ECG) is a basic test for screening cardiovascular disease. However, the impact of ECG abnormalities on cardiovascular prognosis in patients with CKD is largely unknown. A total of 2442 patients with CKD (stages 3–5) who underwent ECG between 2013 and 2015 were selected from the electronic health record database of the Korea University Anam Hospital. ECG abnormalities were defined using the Minnesota classification. The five-year major adverse cerebrocardiovascular event (MACCE), the composite of death, myocardial infarction (MI), and stroke were analyzed. The five-year incidences for MACCE were 27.7%, 20.8%, and 17.2% in patients with no, minor, and major ECG abnormality (*p* < 0.01). Kaplan–Meier curves also showed the highest incidence of MI, death, and MACCE in patients with major ECG abnormality. Multivariable Cox regression analysis revealed age, sex, diabetes, CKD stage, hsCRP, antipsychotic use, and major ECG abnormality as independent risk predictors for MACCE (adjusted HR of major ECG abnormality: 1.39, 95% CI: 1.09–1.76, *p* < 01). Among the detailed ECG diagnoses, sinus tachycardia, myocardial ischemia, atrial premature complex, and right axis deviation were proposed as important ECG diagnoses. The accuracy of cardiovascular risk stratification was improved when the ECG results were added to the conventional SCORE model (net reclassification index 0.07). ECG helps to predict future cerebrocardiovascular events in CKD patients. ECG diagnosis can be useful for cardiovascular risk evaluation in CKD patients when applied in addition to the conventional risk stratification model.

## 1. Introduction

Chronic kidney disease (CKD) has been suggested to be a very strong risk factor for cardiovascular disease (CVD) and increased cardiovascular mortality [1,2,3,4]. Recent US Medicare data demonstrated that the prevalence of heart failure, acute myocardial infarction, and cerebrovascular accident/transient ischemic attack in patients with CKD are two- to four-times higher compared to patients without CKD [5]. Conventional cardiovascular risk factors such as hypertension and diabetes mellitus are very common in patients with CKD and are implicated in higher incidence of CVD and its mortality in patients with CKD [6]. Therefore, the early identification of high-risk CVD patients can lead to targeted strategies to improve cardiovascular prognosis in patients with CKD.

The standard 12-lead electrocardiogram (ECG) is an accessible and inexpensive test and has therefore been widely used for diagnosing or screening CVD. ECG reflects the electrophysiological and structural state of the heart. Previous studies demonstrated that abnormal ECG findings are associated with cardiovascular mortality even in the general population [7,8]. Left ventricular hypertrophy (LVH) diagnosed via ECG is a surrogate marker for target organ damage in hypertensive patients, but other ECG abnormalities are also associated with kidney damage such as microalbuminuria [9]. Several ECG indices such as PR and QT intervals were also reported to be associated with CVD incidence and mortality in patients with CKD [10,11]. However, few studies have been conducted on more than 1000 patients with CKD, especially in Asian people.

Here, we explored the demographic characteristics according to ECG abnormality and compared their cerebrocardiovascular prognostic impacts.

## 2. Materials and Methods

### 2.1. Study Design

The present study was a retrospective cohort study using the electronic health record database of the Korea University Anam Hospital in Korea. For the study population selection, patients who underwent ECG between January 2013 and December 2015 were selected. Patients with missing clinical and laboratory data (e.g., serum creatinine level) within six months of ECG acquisition and patients without CKD with more than a 60 mL/min/1.73 m² of Modification of Diet in Renal Disease (MDRD) glomerular filtration rate (GFR) were excluded. Finally, 2442 patients with CKD remained for further analysis.

### 2.2. Standardization of Computerized ECG Diagnosis

ECG machines automatically generated ECG diagnoses and ancillary descriptions through the approved computerized algorithm. The performance of computerized ECG diagnosis is known to be comparable to physicians’ interpretations [12,13,14]. Automated ECG interpretation is also cost- and time-effective with less intra- and inter-observer variability. For the present study, the computerized ECG diagnoses were transformed to the terminology of SNOMED CT [15]. SNOMED CT mapping for ECG diagnosis was performed using web-based software, which is an integrated algorithm using cosine similarity and rule-based hierarchy (available at cdal.korea.ac.kr/ECG2CDM). Its conversion accuracy is 99.9%. Then, the computerized ECG diagnoses were further categorized using the Minnesota code classification [16]. For patients with multiple ECG results, the earliest ECG results were selected. Each patient was grouped into the categories of major, minor, or no ECG abnormality according to the Minnesota code classification among different ECG diagnoses. The major ECG abnormalities include major Q-wave abnormalities, minor Q-wave abnormalities plus ST-T abnormalities, major isolated ST-T abnormalities, complete or intermittent left bundle-branch block (LBBB)/right bundle-branch block (RBBB), non-specific intraventricular block, RBBB with left anterior hemiblock, the Brugada pattern, LVH plus ST-T abnormalities, QT prolongation, atrial fibrillation or flutter, major AV conduction abnormalities, ventricular fibrillation or asystole, and supraventricular tachycardia. The minor ECG abnormalities include minor isolated Q/QS waves, minor ST/T abnormalities, high R waves, ST segment elevation, incomplete LBBB/RBBB, a short/long PR interval, left/right axis deviation, premature beats, a wandering atrial pacemaker, sinus tachycardia, sinus bradycardia, persistent supraventricular rhythm, low-voltage QRS, a high-amplitude *p* wave, left atrial enlargement, fragmented QRS, and early repolarization. Each ECG abnormality was defined according to the Minnesota code manual [16].

### 2.3. Definitions and Study Endpoint

Patients with hypertension were defined as being on anti-hypertensive medication or having diagnosis codes I10–15 of the ICD-10 codes. Patients with diabetes mellitus were defined as having HbA1c ≥ 6.5%, being on antidiabetic medication, or having diagnosis codes E10–E14 of the ICD-10 codes. Patients with dyslipidemia were defined as having a statin or ezetimibe prescription, total serum cholesterol ≥ 240 mg/dL, low-density lipoprotein cholesterol ≥ 160 mg/dL, triglyceride ≥ 200 mg/dL, or high-density lipoprotein cholesterol < 40 mg/dL. Patients on dialysis were defined as having diagnosis codes for end-stage renal failure on dialysis or procedure codes for dialysis and dialysis care education. Patients with Parkinson’s disease and epilepsy were defined as having respective diagnoses codes. Medication which could affect ECG profiles were classified into 7 categories (*β*-blockers (type II antiarrhythmics), non-dihydropyridine calcium channel blockers (non-DHP CCB; type IV antiarrhythmics), other antiarrhythmics, antidepressants and antipsychotics, prokinetics, antiepileptics, and other medications; Table A1). Cardiovascular risk was calculated using the Systemic Coronary Risk Evaluation (SCORE) model and classified as low-to-moderate, high, and very high [17,18,19].

The primary endpoints of this study were a five-year major adverse cerebrocardiovascular event (MACCE), a composite of death, new-onset myocardial infarction (MI), and stroke. MI was defined as having an OMOP-CDM concept ID for MI or a serum CK-MB level greater than upper limit of normal with a rising and/or falling pattern. Stroke was defined as having the corresponding OMOP-CDM concept ID or having acute, sub-acute or recent cerebral infarction findings on a brain MRI. Survival time was from the follow-up start date (date of the earliest ECG) to the date of MACCE or until the end of the follow-up, whichever came first.

### 2.4. Statistical Analysis

Baseline characteristics are shown as the mean ± SD or *n* (%). The chi-square test and Analysis of Variance (ANOVA) were used to compare the categorical variables and continuous variables between groups. The probabilities for MACCE and the other endpoints were calculated usinb the Kaplan–Meier curves and compared using the log-rank test. Multivariable Cox proportional hazards regression analysis was performed to evaluate the relationship between ECG result group and MACCE risk. ECG diagnoses for multivariable analysis were selected when their prevalence was more than 1% and the *p*-value of univariate analysis was less than 0.1. Clinical risk factors were selected when the *p*-value of univariate analysis was less than 0.1. Among the selected ECG features, the multivariable Cox proportional hazards regression models were fitted with a backward elimination approach that satisfied a significance level of 0.05. The discrimination of the models was assessed using c-statistics and the net reclassification index (NRI) [20,21,22]. The proportional hazards assumption for the variables in the models were assessed by inspecting the Schoenfeld residuals. All analyses were performed using the SAS 9.4 (SAS Institute Inc., Cary, NC, USA) program and the R program (Version 3.6.1).

## 3. Results

### 3.1. Baseline Characteristics

The baseline characteristics of the patients are described in Table 1. The numbers of patients with major, minor, and no ECG abnormality were 921 (37%), 649 (27%), and 872 (36%). Patients with major abnormal ECG results were older and the proportions of hypertension, diabetes mellitus, and dyslipidemia were also higher compared with patients with no or minor ECG abnormalities. The proportions of dialysis patients were also higher in the group of patients with a major ECG abnormality than the other two groups (8.1%, 8.3%, and 12.7%, *p* < 0.01). The proportions of patients who had experienced myocardial infarction were higher in patients with major ECG abnormality than in the others, respectively (3.4%, 7.2%, 16.1%, *p* < 0.01). The proportions of patients taking beta blockers, non-DHP-CCB, and other antiarrhythmics were higher in patients with major ECG abnormality than in the others, respectively (22.0%, 28.0%, and 41.4%, *p* < 0.01 for beta blocker; 3.7%, 5.9%, and 8.3%, *p* < 0.01 for non-DHP-CCB; 1.0%, 2.6%, and 9.7%, *p* < 0.01 for other antiarrhythmics). The laboratory findings showed higher serum creatinine and hsCRP levels in patients with a major ECG abnormality than in the others. The proportion of the very high-risk group in the 10-year CVD risk estimate (SCORE) was also higher in patients with a major ECG abnormality than in the others (39.6%, 50.9%, and 54.6%, *p* < 0.01).

### 3.2. Proportion of ECG Abnormalities

The three most common ECG diagnoses of major ECG abnormality were a prolonged QT interval, AV block (first degree), and an abnormal T wave (lateral leads) (Table A2). An abnormal T wave was the most common abnormal ECG diagnosis in CKD III/IV, and a QT interval (prolonged) was the most common abnormal ECG diagnosis in CKD V. These were followed by sinus bradycardia in CKD III/IV and an abnormal T wave in CKD V (Table 2). Of note, the prevalence of a prolonged QT interval increased as the CKD stage increased (5.8% in CKD III, 9.2% in CKD IV, and 20.7% in CKD V).

### 3.3. ECG Abnormality and MACCE

**Figure 1:**
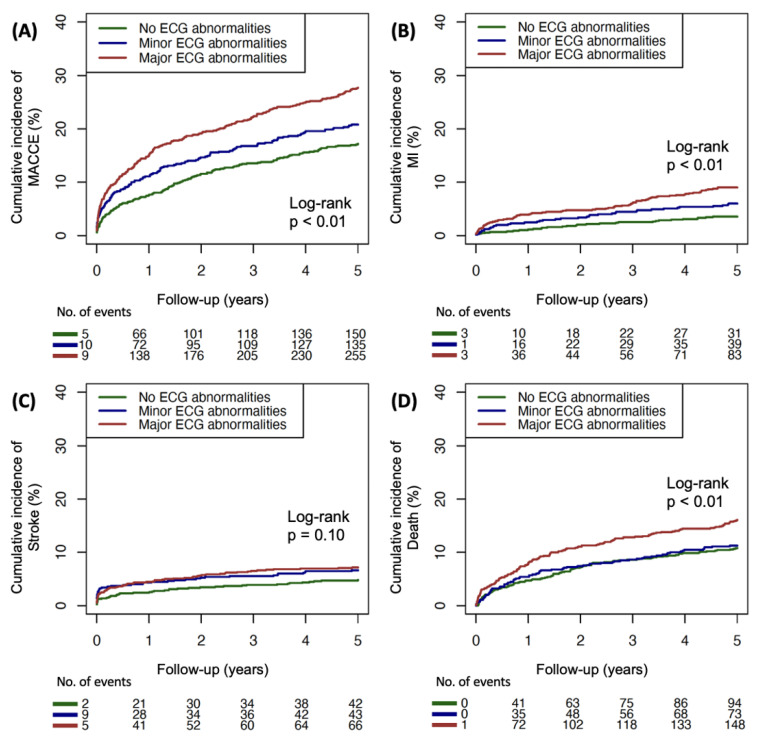
Kaplan–Meier plot of cumulative incidence of (**A**) MACCE, (**B**) MI, (**C**) stroke, and (**D**) death according to ECG abnormality.

A total of 2442 patients were analyzed to evaluate the clinical impact of ECG abnormality on MACCE in patients with CKD. The median follow-up period was 1826 days. The cumulative incidence of MACCE is shown in Table 3. The incidence rate of MACCE in the group with major ECG abnormality was higher compared with that in the other groups (17.2%, 20.8%, and 27.7%, *p* < 0.01). Among MACCE, new-onset MI, stroke, and all-cause death showed higher incidence in the group with major ECG abnormalities. The Kaplan–Meier plots of cumulative incidence of MACCE are shown in Figure 1A–D. The incidence of MACCE, MI, and all-cause death was higher in the major ECG abnormality group, but stroke was not statistically significant.

### 3.4. Multivariable Cox Regression Analysis

Cox regression analysis was performed to evaluate whether ECG abnormality was a meaningful risk factor for MACCE occurrence after adjusting for various confounding factors (Table 4). It proposed major ECG abnormalities for the independent risk factor for MACCE in patients with CKD (HR: 1.39, 95% CI: 1.09–1.76, *p* < 0.01). The other independent risk factors were age, male sex, diabetes mellitus, CKD stage 5, hsCRP, and antiepileptic use.

There were a total of 70 detailed ECG diagnoses in the study population. Twenty-eight ECG diagnoses show a prevalence of more than 1% (Table A2). Eleven abnormal ECG diagnoses (sinus tachycardia, abnormal T wave (lateral leads), QT prolongation, abnormal Q wave (inferior leads), atrial premature complex, abnormal T wave, right axis deviation, left atrial enlargement, abnormal Q wave (anterior leads), sinus arrhythmia, and sinus bradycardia) had a *p*-value of less than 0.1 for univariate analysis (Table A3). Multivariable analysis considering these eleven ECG diagnoses proposed four ECG diagnoses (sinus tachycardia, abnormal T wave (lateral leads), atrial premature complex, and right axis deviation) as the independent predictors for MACCE (Table 4).

The net reclassification index and c-statistics were analyzed by adding the ECG result to the SCORE risk assessment model to evaluate whether it improves the predictive power for major cardiovascular events (Table 5). The ECG result was applied using two approaches. One was the SCORE model combined with the ECG abnormality category, and the other was the SCORE model combined with the detailed ECG diagnosis (4 ECG diagnoses chosen using multivariable analysis). Both models showed higher c-statistics (c-statistic: 0.59, 95% CI: 0.57–0.61 for SCORE model combined with ECG abnormality; c-statistic: 0.59, 95% CI: 0.57–0.61 for SCORE model with ECG diagnosis) compared to the original SCORE model (c-statistic: 0.57, 95% CI: 0.54–0.59). Additionally, the new model adopting ECG diagnoses significantly improved the net reclassification index (0.07, 95% CI: 0.02–0.12 for the SCORE model combined with ECG abnormality; 0.07, 95% CI: 0.02–0.13 for the SCORE model with ECG diagnosis).

## 4. Discussion

This study is the first long-term observational study to explore the prevalence and clinical impact of ECG abnormality on cerebrocardiovascular prognosis in Asian patients with CKD. We found several clinical perspectives on ECG with respect to cerebrocardiovascular prognosis in patients with CKD. ECG is the most fundamental test for cardiovascular disease and could be used to evaluate the risk of cardiovascular disease, especially in CKD patients. In addition, ECG could detect subclinical cardiovascular disease. Although ECG is not included in patient evaluation in the current CKD guidelines, ECG is already widely used as a basic test to evaluate CKD patients in actual clinical practice. 

Several surrogate markers, such as the albumin–creatinine ratio (ACR), pulse wave velocity, and carotid ultrasound, have been proposed for predicting CVD in patients with CKD [23]. Of note, depending on the patient’s race, there are differences in the association of some surrogate markers with CVD, and their decision criteria are accordingly different. For example, ACR levels are higher and have a stronger association with CVD in Asians compared to Europeans [24]. Previously, several studies demonstrated that ECG abnormalities are associated with poor cardiovascular prognosis in patients with CKD [10,11,25]. Although almost all of these studies have been conducted in Caucasian and Black patients with CKD, our study expands these associations in Asian patients with CKD.

As noted above, abnormal T wave, sinus bradycardia, and LVH were the most common ECG abnormalities, and a prolonged QT interval was the most common major ECG abnormality in CKD patients. Abnormal T waves are flat or slightly inverted T waves that may be associated with myocardial ischemia, but in many cases, they may be nonspecific changes associated with LVH or prolonged QT, changes in sympathetic tone, etc. Sinus bradycardia is common in the elderly and may be more frequent in this study group because it is associated with the use of beta-blockers. LVH is known to be a common ECG abnormality among patients with CKD and has been reported to account for nearly one-third of CKD patients. The presence of LVH is an independent predictor of survival in patients with CKD [6] and LVH in end-stage renal disease is an independent risk factor for all-cause and cardiac mortality [26]. However, in the univariate analysis of our study, LVH did not show a meaningful association with MACCE. Severe LVH is accompanied by ST-T change and is also associated with clinical features such as age, diabetes, and CKD, so the effect of LVH alone on MACCE occurrence may not be as great as before. Therefore, it is reasonable to consider other ECG diagnoses rather than LVH alone for the occurrence of MACCE.

The QT interval reflects both the conduction and repolarization of the heart and is affected by electrolyte imbalance as well as myocardial ischemic condition. Previous studies reported that the QT interval is associated with cardiovascular disease, including myocardial infarction and deaths [10,25,27,28,29,30,31]. Similarly, our univariable analysis revealed the increased HR of prolonged QT interval for MACCE. However, a prolonged QT interval did not remain an independent risk predictor in multivariable Cox regression analysis. This is thought to be due to the high rate of use of antipsychotic drugs with a QT-prolonging effect in patients with severe CKD (14.3% in total patients with CKD), so a prolonged QT interval was excluded from the variable selection process of the multivariable Cox regression analysis.

The multivariable Cox regression analysis proposed four ECG diagnoses (sinus tachycardia, abnormal T wave (lateral leads), atrial premature complex, and right axis deviation) as independent risk predictors for MACCE. In a previous study, Palatini et al. showed that tachycardia was an independent predictor of MACCE among hypertensive patients and concluded that the measurement of HR should add to risk stratification for MACCE and mortality [32]. Although isolated premature atrial contractions were not associated with an increased risk of sudden cardiac death, they were associated with cardiac-related and all-cause mortality [33,34]. Our result was compatible with these previous studies. However, in case of right axis deviation, Yuta Seko et al. showed that left axis deviation was associated with a higher risk of MACE and all-cause death, but right axis deviation was not [35]. However, it was found that right ventricular dysfunction was strongly associated with CKD and poor prognosis in chronic systolic HF patients [36]. Traditionally, right axis deviation commonly reflects right-heart disease. In detail, right ventricular hypertrophy and right axis deviation suggest that patients have the condition of right-side overload, which is usually caused by pressure (e.g., pulmonary hypertension) or volume overload. In this regard, transthoracic echocardiography to evaluate right-side heart function may be necessary if ECG shows unexpected right axis deviation in a CKD patient. Further research should be performed on this whether right-side deviation is truly associated with right ventricular dysfunction or not, and on its association with MACCE and all-cause death in patients with CKD.

It is known that all-cause mortality is substantially higher in dialysis (15–20% at 1 year) than in heart failure or post-infarction patients (3–8% at 1 year) [37]. Since, all-cause mortality in dialysis patients is higher than in heart failure or post-infarction patients, it is hard to explain the high SCD rate among dialysis patients by heart failure and infarction only. SCD in patients with CKD has a complex mechanism and, at present, there is no SCD-specific risk factor. As noted above, four ECG diagnoses were related to MACCE. Especially in the case of atrial premature complex, it does not have a direct relationship with heart failure and infarction. Thus, we cautiously suggest that atrial premature complexes might have a relationship with SCD in patients with CKD. Future studies need to investigate the reproducibility of our result and underline the mechanism of SCD.

Patients with CKD not only show an increased risk of sudden cardiac death, but also have clearly different pathophysiology and causes of sudden cardiac death compared to the general population [6,38]. For this reason, patients with CKD need a more specific risk stratification model compared to general population. Regardless of the detailed ECG diagnosis or ECG categories, when the ECG results were additionally applied to the clinical cardiovascular risk assessment model, it further reclassified a small but not insignificant 7.2% of CKD patients. This number might be small, but it represents significant progress and could be adopted to develop a better cardiovascular risk assessment model in the future.

There are several limitations in our study. First, we conducted our study using automated ECG diagnosis provided by a machine. There is a possibility of a wrong interpretation being generated, because the machine did not consider the clinical information. Second, each ECG abnormality category contained many ECG diagnoses that do not share a common pathophysiology. It is difficult to apply the ECG abnormality category as a decision-making factor. There are limitations to directly applying the results of this study to actual clinical practice. However, since ECG abnormality is significantly related to the occurrence of MACCE, ECG could play a clinically important role in CKD patients. Further studies may develop the methods to estimate the cardiovascular risk group of CKD patients more accurately. This could provide important information for the screening of patients who need aspirin or a statin for primary and secondary prevention of cardiovascular disease, or for the screening of patients who need an additional work-up for subclinical cardiovascular disease in the future. Third, since different ECG abnormality criteria can be used for each study, we should be careful when comparing or applying our study to other studies. Some previous studies adopted different criteria for major and minor ECG abnormality [39,40,41]. Although the Minnesota code classification, which was adopted in our study, cannot be considered the only standard for the classification of ECG abnormalities, we would like to present reproducible and clinically applicable results in other institutions by utilizing standardized ECG diagnosis and classification criteria. Additionally, we applied the latest version of the Minnesota code classification. This is the first study to use the updated version. Fourth, since we defined diseases largely based on diagnostic codes, if the diagnostic code was entered incorrectly, there is a possibility that some data would was missed. Fourth, only 639 (26.4%) patients with advanced CKD under eGFR 30 mL/min/1.73 m² were included in our study. Similarly, the proportion of patients with severe CKD was about 5–20% in previous studies [10,11,25].

In conclusion, major ECG abnormalities in Asian patients with CKD are associated with cerebrocardiovascular events, especially MI and all cause-death. Further research is needed on more precise cerebrocardiovascular risk assessment and appropriate intervention strategies using ECG in the future.

## 5. Conclusions

In conclusion, major ECG abnormalities in Asian patients with CKD are associated with cerebrocardiovascular events, especially MI and all-cause death. Further research is needed on more precise cerebrocardiovascular risk assessment and appropriate intervention strategies using ECG in the future.

## Figures and Tables

**Table 1:** Baseline characteristics of study groups according to ECG abnormality.

	Total	No ECG	Minor ECG	Major ECG	*p*-Value
	Population	Abnormality	Abnormality	Abnormality	
	(*n* = 2442)	(*n* = 872)	(*n* = 649)	(*n* = 921)	
Age, years	70.7 ± 12.9	68.0 ± 12.7	70.6 ± 13.1	73.2 ± 12.4	<0.01
Male	1211 (49.6)	395 (45.3)	339 (52.2)	477 (51.8)	<0.01
Body mass index	24.7 ± 4.1	24.7 ± 3.7	24.4 ± 4.7	24.9 ± 4.1	0.05
Current smoker	528 (21.6)	184 (21.1)	158 (24.4)	186 (20.2)	0.13
Alcohol drinking	524 (21.5)	186 (21.3)	158 (24.4)	180 (19.5)	0.07
Hypertension	1584 (64.9)	521 (59.8)	386 (59.5)	677 (73.5)	<0.01
Anti-hypertensive Medication	1519 (62.2)	501 (57.5)	372 (57.3)	646 (70.1)	<0.01
Diabetes mellitus	1270 (52.0)	437 (50.1)	305 (47.0)	528 (57.3)	<0.01
Oral hypoglycemic agent	635 (26.0)	216 (24.8)	148 (22.8)	271 (29.4)	<0.01
Insulin use	498 (20.4)	167 (19.2	104 (16.0)	227 (24.7)	<0.01
Dyslipidemia	1497 (61.3)	502 (57.6)	375 (57.8)	620 (67.3)	<0.01
Lipid-lowering medication	1067 (43.7)	356 (40.8)	246 (37.9)	465 (50.5)	<0.01
Chronic kidney disease					
Stage III	1803 (73.8)	663 (76.0)	487 (75.0)	653 (70.9)	0.07
Stage IV	238 (9.8)	78 (8.9)	67 (10.3)	93 (10.1)	
Stage V	401 (16.4)	131 (15.0)	95 (14.6)	175 (19.0)	
Dialysis	242 (9.9)	71 (8.1)	54 (8.3)	117 (12.7)	<0.01
Myocardial infarction	225 (9.2)	30 (3.4)	47 (7.2)	148 (16.1)	<0.01
Stroke	316 (12.9)	95 (10. 9)	86 (13.3)	135 (14.7)	0.06
Parkinson’s	35 (1.4)	11 (1.3)	12 (1.9)	12 (1.3)	0.58
Epilepsy	11 (0.5)	3 (0.3)	2 (0.3)	6 (0.7)	0.51
Use of the potential medications affecting ECG					
*β*-blockers	755 (30.9)	192 (22.0)	182 (28.0)	381 (41.4)	<0.01
Non-DHP CCB	146 (6.0)	32 (3.7)	38 (5.9)	76 (8.3)	<0.01
Other antiarrhythmics	115 (4.7)	9 (1.0)	17 (2.6)	89 (9.7)	<0.01
Antidepressants and antipsychotics	350 (14.3)	118 (13.5)	87 (13.4)	145 (15.7)	0.30
Antiepileptics	494 (20.2)	180 (20.6)	109 (16.8)	205 (22.3)	0.03
Prokinetics	454 (18.6)	166 (19.0)	108 (16.6)	180 (19.5)	0.32
Other medications	171 (7.0)	31 (3.6)	42 (6.5)	98 (10.6)	<0.01
Laboratory findings					
Total cholesterol (mg/dL)	162.9 ± 48.5	166.9 ± 49.8	162.8 ± 48.3	159.2 ± 47.1	<0.01
LDL cholesterol (mg/dL)	104.1 ± 35.8	105.4 ± 36.6	105.8 ± 34.4	102.1 ± 35.6	0.16
HDL cholesterol (mg/dL)	42.2 ± 12.5	42.9 ± 12.6	41.6 ± 12.6	42.0 ± 12.3	0.3
Triglyceride (mg/dL)	146.9 ± 101.5	153.5 ± 106.3	152.3 ± 95.6	138.2 ± 100.5	0.01
Fasting glucose (mg/dL)	144.3 ± 75.1	144.1 ± 81.6	138.6 ± 67.2	148.5 ± 73.8	0.04
Hba1c (%)	6.9 ± 1.8	7.0 ± 1.8	6.9 ± 1.7	6.9 ± 1.8	0.89
Creatinine (mg/dL)	2.5 ± 2.8	2.5 ± 2.9	2.4 ± 2.4	2.7 ± 3.0	0.02
hsCRP (mg/dL)	2.6 ± 2.5	2.3 ± 2.4	2.5 ± 2.6	2.9 ± 2.6	<0.01
10-year CVD risk (SCORE)					<0.01
Low/moderate	679 (27.8)	306 (35.1)	177 (27.2)	196 (21.3)	
High	585 (24.0)	221 (25.3)	142 (21.9)	222 (24.1)	
Very high	1178 (48.2)	345 (39.6)	330 (50.9)	503 (54.6)	

Values are presented as *n* (%) or mean ± standard deviation. LDL—low-density lipoprotein; CVD—cardiovascular; HDL—high-density lipoprotein; hsCRP—high-sensitivity C-reactive protein; non-DHP CCB—non-dihydropyridine calcium channel blocker.

**Table 2:** Top 3 ECG diagnoses according to CKD stage and ECG group.

CKD Stage	Minnesota Code Classification	ECG Diagnosis (SNOMED)	No. (%)
III	No ECG abnormalities	Normal sinus rhythm/sinus rhythm	1290 (71.6)
		Sinus arrhythmia	32 (1.8)
		Aberrant conduction complex	28 (1.6)
	Minor ECG abnormalities	Abnormal T wave	284 (15.8)
		Sinus rhythm (bradycardia)	249 (13.8)
		LVH	161 (8.9)
	Major ECG abnormalities	AV block (1st degree)	118 (6.5)
		Abnormal T wave (lateral leads)	114 (6.3)
		QT interval (prolonged)	105 (5.8)
IV	No ECG abnormalities	Normal sinus rhythm/sinus rhythm	175 (73.5)
		Sinus arrhythmia	6 (2.5)
		Aberrant conduction complex	13(1.3)
	Minor ECG abnormalities	Abnormal T wave	37 (15.6)
		Sinus rhythm (bradycardia)	30 (12.6)
		Left axis deviation	26 (10.9)
	Major ECG abnormalities	QT interval (prolonged)	22 (9.2)
		AV block (1st degree)	16 (6.7)
		Abnormal T wave (lateral leads)	16 (6.7)
V	No ECG abnormalities	Normal sinus rhythm/Sinus rhythm	334 (83.3)
		Sinus arrhythmia	4 (1.0)
	Minor ECG abnormalities	Abnormal T wave	66 (16.5)
		LVH	63 (15.7)
		Sinus rhythm (bradycardia)	26 (6.5)
	Major ECG abnormalities	QT interval (prolonged)	83 (20.7)
		Abnormal T wave (lateral leads)	31 (7.7)
		AV block (1st degree)	28 (7.0)

Values are presented as *n* (%). AV—atrioventricular; CKD—chronic kidney disease; LVH—left ventricular hypertrophy.

**Table 3:** The cumulative incidence rates of new-onset MI, stroke, death, and MACCE.

	Total	No ECG	Minor ECG	Major ECG	*p*-Value
	Population	Abnormality	Abnormality	Abnormality	
	(*n* = 2442)	(*n* = 872)	(*n* = 649)	(*n* = 921)	
New-onset MI	153 (6.3)	31 (3.6)	39 (6.0)	83 (9.0)	<0.01
New-onset stroke	151 (6.2)	42 (4.8)	43 (6.6)	66 (7.2)	0.04
All-cause death	315 (13.0)	94 (10.8)	73 (11.3)	148 (16.1)	<0.01
MACCE	540 (22.3)	150 (17.2)	135 (20.8)	255 (27.7)	<0.01

Values are presented as proportion of incidence (%). MI—myocardial infarction; MACCE—major adverse cerebrocardiovascular event.

**Table 4:** Multivariable Cox regression analyses for MACCE.

Risk Factor	HR (95% CI)	
	Unadjusted	Adjusted
A. Using the ECG abnormality categories of the Minnesota ECG classification		
Age, years	1.01 (1.01–1.02) **	1.01 (1.01–1.02) **
Male	1.26 (1.06–1.49) **	1.26 (1.03–1.53) *
Diabetes mellitus	1.51 (1.27–1.79) **	1.33 (1.08–1.65) **
CKD stage		
III	Reference	Reference
IV	1.34 (1.02–1.75) *	1.27 (0.93–1.73)
V	1.33 (1.08–1.65) **	1.35 (1.04–1.76) *
hsCRP (mg/dL)	1.09 (1.06–1.13) **	1.08 (1.04–1.11) **
Antiepileptics	1.54 (1.27–1.86) **	1.43 (1.15–1.78) **
ECG abnormality		
Normal	Reference	Reference
Minor	1.25 (0.99–1.58)	1.12 (0.85–1.48)
Major	1.73 (1.41–2.12) **	1.38 (1.09–1.76) **
B. Using the detailed ECG diagnosis		
Age, years	1.01 (1.01–1.02) **	1.02 (1.01–1.03) **
Male	1.26 (1.06–1.49) **	1.33 (1.09–1.63) **
Diabetes mellitus	1.51 (1.27–1.79) **	1.33 (1.08–1.65) **
CKD stage		
III	Reference	Reference
IV	1.34 (1.02–1.75) *	1.29 (0.95–1.76)
V	1.33 (1.08–1.65) **	1.39 (1.07–1.80) *
hsCRP (mg/dL)	1.09 (1.06–1.13) **	1.07 (1.03–1.11) **
Antiepileptics	1.54 (1.27–1.86) **	1.45 (1.16–1.81) **
ECG diagnoses		
Sinus rhythm (tachycardia)	1.95 (1.43–2.66) **	2.13 (1.46–3.10) **
Abnormal T wave (lateral leads)	1.69 (1.27–2.24) **	1.81 (1.31–2.50) **
Atrial premature complex	1.96 (1.21–3.18) **	1.84 (1.06–3.21) *
Right axis deviation	1.95 (1.13–3.38) *	2.20 (1.04–4.66) *

* Significant at *p* < 0.05, ** Significant at *p* < 0.01. Values are presented as hazard ratio (95% confidence interval). CKD—chronic kidney disease; hsCRP—high-sensitivity C-reactive protein.

**Table 5:** Model comparison of the SCORE model alone and of the SCORE model with ECG abnormality or detailed diagnosis.

	SCORE Model	SCORE Model + ECG Abnormality	SCORE Model + ECG Diagnosis
C-statistics (95% CI)	0.57 (0.54–0.59)	0.59 (0.57–0.61)	0.59 (0.57–0.61)
NRI (95% CI)	Reference model	0.07 (0.02–0.12)	0.07 (0.02–0.13)

NRI—net reclassification index. SCORE model: sex + systolic blood pressure + current smoker + total cholesterol. ECG abnormality included minor and major ECG abnormality. ECG diagnosis included sinus rhythm (tachycardia), myocardial ischemia (lateral), atrial premature complex, and right axis deviation.

## Data Availability

Not applicable.

## Appendix A

**Table A1:** List of the potential medications affecting ECG result.

Category	Drug	
*β*-blocker (Type II antiarrhythmics)	Propranolol, Acebutolol, Labetalol, Oxprenolol, Metoprolol, Nadolol, Atenolol, Propranolol, Pindolol, Nebivolol, Bisoprolol, Carvedilol	
Non-DHP CCB (Type IV antiarrhythmics)	Verapamil, Diltiazem	
Other Antiarrhythmics	Type IA antiarrhythmics	Quinidine, Procainamide, Disopyramide
	Type IC antiarrhythmics	Flecainide, Encainide
	Type III antiarrhythmics	Sotalol, Amiodarone
	Others	Digoxin, Ivabradine
Antidepressants and antipsychotics	Tricyclic antidepressants	Amitriptyline, Doxepin, Imipramine, Nortriptyline, Desipramine
	Other antidepressants	Mianserin, Citalopram, Escitalopram, Venlafaxine, Bupropion, Moclobemide
	Antipsychotics	Chlorpromazine, Haloperidol, Droperidol, Quetiapine, Olanzapine, Amisulpride, Thioridazine
Prokinetics	Dopaminergic D_2_-Antagonist drugs	Metoclopramide, Domperidone
	Serotonergic 5-HT_4_ agonist drugs	Cisapride, Mosapride, Prucalopride, Tegaserod
Antiepileptics	Gabapentin, Pregabalin, Retigabine, Carbamazepine, Lamotrigine, Oxcarbazepine, Phenytoin, Clonazepam, Diazepam, Phenobarbital, Levetiracetam, Topiramate, Valproate	
Other medications	Antihistamines	Diphenhydramine, Astemizole, Loratadine, Terfenadine
	Macrolides	Erythromycin, Clarithromycin
	Others	Chloroquine, Hydroxychloroquine

Non-DHP CCB—non-dihydropyridine calcium channel blocker.

**Table A2:** Prevalence of ECG diagnosis (prevalence > 1%).

ECG Diagnosis	*n* (%)
Normal sinus rhythm/sinus rhythm	1799 (73.7)
Abnormal T wave	387 (15.9)
Sinus rhythm (bradycardia)	305 (12.5)
LVH	243 (10.0)
QT interval (prolonged)	210 (8.6)
AV block (1st degree)	162 (6.6)
Abnormal T wave (lateral leads)	161 (6.6)
Left axis deviation	141 (5.8)
Abnormal Q wave (inferior leads)	132 (5.4)
RBBB	124 (5.1)
Atrial fibrillation	122 (5)
Sinus rhythm (tachycardia)	119 (4.9)
ST-T abnormality (non-specific)	106 (4.3)
Abnormal T wave (anterior leads)	73 (3.0)
Voltage (decreased)	59 (2.4)
Abnormal Q wave (septal leads)	57 (2.3)
Ventricular premature complex	54 (2.2)
Atrial premature complex	45 (1.8)
Abnormal T wave (inferior leads)	45 (1.8)
Sinus arrhythmia	42 (1.7)
P wave (left atrial enlargement)	40 (1.6)
Wide QRS complex	39 (1.6)
Abnormal Q wave (anterior leads)	36 (1.5)
Right axis deviation	34 (1.4)
Aberrant conduction complex	31 (1.3)
Atrial fibrillation (rapid ventricular response)	31 (1.3)
LAFB	29 (1.2)
Abnormal Q wave (anteroseptal leads)	29 (1.2)

AV—atrioventricular; LAFB—left anterior fascicular block; LVH—left ventricular hypertrophy; RBBB—right bundle-branch block.

**Table A3:** Univariable Cox regression analysis for MACCE.

	HR (95% CI)	*p* Value
No ECG abnormality	Reference	
Minor ECG abnormality	1.25 (0.99–1.58)	0.06
Major ECG abnormality	1.73 (1.41–2.12)	<0.01
Age	1.01 (1.01–1.02)	<0.01
Sex	1.26 (1.06–1.49)	<0.01
Smoking	1.13 (0.92–1.38)	0.24
Drinking	0.85 (0.68–1.05)	0.13
Hypertension	1.38 (1.14–1.66)	<0.01
Diabetes	1.51 (1.27–1.79)	<0.01
Dyslipidemia	1.43 (1.19–1.71)	<0.01
Parkinson	0.63 (0.26–1.53)	0.31
Epilepsy	0.37 (0.05–2.63)	0.32
CKD stage 3	Reference	
CKD stage 4	1.34 (1.02–1.75)	0.03
CKD stage 5	1.33 (1.08–1.65)	<0.01
hsCRP (mg/dL)	1.09 (1.06–1.13)	<0.01
*β*-blocker	1.29 (1.08–1.54)	<0.01
Non-DHP CCB	1.22 (0.88–1.70)	0.23
Antiarrhythmics	1.06 (0.72–1.57)	0.75
Antipsychotics	1.47 (1.19–1.82)	<0.01
Antiepileptics	1.54 (1.27–1.86)	<0.01
Prokinetics	1.45 (1.19–1.77)	<0.01
Other medications	1.47 (1.10–1.96)	<0.01
ECG diagnosis		
Sinus rhythm (tachycardia)	1.95 (1.43–2.66)	<0.01
Abnormal T wave (lateral leads)	1.69 (1.27–2.24)	<0.01
QT interval (prolonged)	1.50 (1.15–1.95)	<0.01
Abnormal Q wave (inferior leads)	1.60 (1.17–2.20)	<0.01
Atrial premature complex	1.96 (1.21–3.18)	<0.01
T wave (abnormal)	1.34 (1.08–1.66)	<0.01
Normal sinus rhythm / sinus rhythm	0.79 (0.66–0.95)	0.01
Right axis deviation	1.95 (1.13–3.38)	0.02
P wave (left atrial enlargement)	1.83 (1.07–3.11)	0.03
Abnormal Q wave (anterior leads)	1.77 (1.02–3.07)	0.04
Sinus arrhythmia	1.68 (0.99–2.86)	0.05
Sinus rhythm (bradycardia)	0.79 (0.60–1.04)	0.09
Atrial fibrillation (rapid ventricular response)	1.68 (0.90–3.14)	0.10
Abnormal T wave (inferior leads)	1.51 (0.89–2.57)	0.13
Aberrant conduction complex	1.57 (0.84–2.94)	0.16
Wide QRS complex	1.49 (0.84–2.65)	0.17
Atrial fibrillation	1.25 (0.88–1.79)	0.22
Abnormal T wave (anterior leads)	1.30 (0.83–2.04)	0.25
Left axis deviation	1.21 (0.86–1.70)	0.27
Ventricular premature complex	1.30 (0.78–2.18)	0.31
ST-T abnormality (non-specific)	1.22 (0.83–1.79)	0.32
LVH	1.13 (0.86–1.48)	0.37
Voltage (decreased)	1.20 (0.72–2.01)	0.48
Abnormal Q wave (anteroseptal leads)	1.27 (0.63–2.56)	0.50
RBBB	1.10 (0.76–1.58)	0.63
Abnormal Q wave (septal leads)	1.14 (0.67–1.93)	0.64
LAFB	0.91 (0.41–2.04)	0.82
AV block (1st degree)	0.97 (0.69–1.36)	0.84

AV—atrioventricular; hsCRP—high-sensitivity C-reactive protein; non-DHP CCB—non-dihydropyridine calcium channel blocker; LAFB—left anterior fascicular block; LVH—left ventricular hypertrophy; RBBB—right bundle-branch block.
